# Identification and Deletion of *Tft1*, a Predicted Glycosyltransferase Necessary for Cell Wall β-1,3;1,4-Glucan Synthesis in *Aspergillus fumigatus*

**DOI:** 10.1371/journal.pone.0117336

**Published:** 2015-02-27

**Authors:** Danial Samar, Joshua B. Kieler, J. Stacey Klutts

**Affiliations:** 1 Department of Pathology, University of Iowa Carver College of Medicine, 200 Hawkins Dr. Iowa City, IA 52242, United States of America; 2 Pathology and Laboratory Medicine, Iowa City VA Health System, 601 Highway 6 West, Iowa City, IA 52246, United States of America; Universidade de Sao Paulo, BRAZIL

## Abstract

*Aspergillus fumigatus* is an environmental mold that causes severe, often fatal invasive infections in immunocompromised patients. The search for new antifungal drug targets is critical, and the synthesis of the cell wall represents a potential area to find such a target. Embedded within the main β-1,3-glucan core of the *A. fumigatus* cell wall is a mixed linkage, β-D-(1,3;1,4)-glucan. The role of this molecule or how it is synthesized is unknown, though it comprises 10% of the glucans within the wall. While this is not a well-studied molecule in fungi, it has been studied in plants. Using the sequences of two plant mixed linkage glucan synthases, a single ortholog was identified in *A. fumigatus* (*Tft1*). A strain lacking this enzyme (*tft1Δ*) was generated along with revertant strains containing the native gene under the control of either the native or a strongly expressing promoter. Immunofluorescence staining with an antibody against β-(1,3;1,4)-glucan and biochemical quantification of this polysaccharide in the *tft1Δ* strain demonstrated complete loss of this molecule. Reintroduction of the gene into the knockout strain yielded reappearance in amounts that correlated with expected expression of the gene. The loss of *Tft1* and mixed linkage glucan yielded no in vitro growth phenotype. However, there was a modest increase in virulence for the *tft1Δ* strain in a wax worm model. While the precise roles for β-(1,3;1,4)-glucan within *A. fumigatus* cell wall are still uncertain, it is clear that *Tft1* plays a pivotal role in the biosynthesis of this cell wall polysaccharide.

## Introduction

Aspergillus fumigatus is a ubiquitous, environmental fungus that is currently the most commonly encountered mold pathogen among severely immunocompromised patients [1]. With advancement in medical therapies that lead to immunosuppression, there has been an increase in the risk of serious, invasive *Aspergillus* infections [2]. This increased risk, coupled with relatively slow diagnostics and ineffective antifungal therapies for advanced infections, often leads to poor outcomes, especially in bone marrow/stem cell transplant patients. New antifungal targets effective against *A. fumigatus* and other mold pathogens are sorely needed. One attractive biological process to target with antifungals is cell wall synthesis, as this structure is essential and unique to fungi. Unfortunately, our understanding of the detailed processes of cell wall synthesis in molds such as *A. fumigatus* is minimal.

The structure of the cell wall of *A. fumigatus* shares a few similarities to the model yeast Saccharomyces cerevisiae. However, major differences exist between the cell walls of these organisms, and inferring mechanisms of cell wall synthesis between yeast and molds has proven challenging. The cell wall of *A. fumigatus* has two major fractions, one that is soluble in alkali and another which is insoluble [3]. The alkali soluble fraction consists primarily of α-1,3 glucan and cell wall associated proteins [3]. The insoluble fraction has cross-linked β-1,3 glucan as its core polysaccharide, with branches of chitin, galactomannan, and a unique mixed linkage β-D-1,3;1,4-glucan [4]. The report of the presence of the β-1,3;1,4-glucan in *A. fumigatus* was the first description of this molecule in fungi [4]. It makes up about 10% of the glucan content in the cell wall of *A. fumigatus*, but its role within the cell wall or in general *Aspergillus* biology is unknown [5]. In contrast, this polysaccharide is a well-studied molecule in plants, where it has importance in the agricultural and food industries [6]. In rice and barley, two genes, *CslF* and *CslH* (‘Cellulose like synthases’), respectively, were shown to encode the cell wall β-1,3;1,4-glucan synthases of these two plants, in which the presence of this cell wall polysaccharide has been shown to be important for cold temperature tolerance [7,8]. Taking the protein sequences for these plant enzymes, we identified a rather distant ortholog within the *A. fumigatus* database (XP_748682), with homology to both plant sequences. We hypothesized that this putative glycosyltransferase was the mixed linkage glucan synthase in *A. fumigatus*. Here, we describe the deletion of the encoding gene in *A. fumigatus* and the analysis of the deletion strain, including growth, virulence and cell wall structural evaluation. We have renamed this gene *Tft1* (*Three Four Transferase* 1), and this represents the first description of a β-1,3;1,4 glucan synthase in fungi.

## Materials and Methods

### Homology searches.

Protein sequences from *CSL-F* and *CSL-H* were used to BLAST the *Aspergillus fumigatus* (Af293) database in NCBI/GenBank. These sequences were aligned with MegAlign (DNASTAR). In addition, the protein sequence of *Tft1* was also used to BLAST search the general NCBI database to determine if orthologs exist in other organisms.

### Growth and culture conditions.

*A. fumigatus* strains were maintained at 37°C on *Aspergillus* minimal media agar (AMM) (http://www.aspergillus.org.uk/indexhome.htm?secure/laboratory_protocols/index.php~main). Hygromycin or phleomycin were added when positive selection was required. Mycelia for analysis were harvested from strains grown in Sabaroud dextrose broth at 37°C with 220 RPM shaking. Conidia for spore inoculum were harvested from MM agar slants grown at 37°C for 4–7 days. 0.1% Tween 80 in 0.01M PBS was used to harvest conidia. Spore concentration was quantified by hemocytometer counts.

### Plasmid and strain generation.

Primer sequences, plasmids and strains used in this study can be found in Tables 1, 2, and 3, respectively. The generation of all strains was via *Agrobacterium tumefaciens* mediated transformation (ATMT) as previously described [9]. Briefly, for *Tft1* deletion, a construct containing the flanking genomic 1kb regions of *Tft1* cloned on either side of a hygromycin resistance marker within the ATMT plasmid pDHT-Y was generated using yeast recombinational cloning of fragments amplified with primers rTFT1-1+ rTFT1-3 and rTFT1-4+ rTFT1-6 off of Af293 genomic DNA; KU80-5+ rTFT1-2 off of pSK485 plasmid; and KU80-4+ rTFT1-L5 off of Topo βrec/HygR plasmid [10]. The resulting plasmid was harvested from yeast and electroporated into A. tumefaciens. The resulting strain was used for co-cultivation with spores from WT *A. fumigatus* (Af293) as previously described [9,11]. Both revertant strains (revtft1(*tft1Δ*::TFT1) and SSA1-revtft1 (*tft1Δ*::SSA1-TFT1) were generated from the *tft1Δ* strain. The revtft1 construct contained upstream 1kb flank of the TFT1 gene with the native TFT1 ORF (TFT1KI-1/TFT1KI-3 off genomic DNA) and the downstream 1kb flank (and TFT1KI-4/TFT1KI-6 off genomic DNA) surrounding the phleomycin resistance cassette (TFT1KI-2/TFT1KI-5 off of pDHT-Y-agtaΔKI). SSA1-Revtft1 was created in similar fashion, with the strong constitutive SSA1 promoter situated between the upstream flank and the TFT1 ORF. The construct contained upstream 1kb flank of the TFT1 gene (TFT1KI-1/Sup-2 off genomic DNA), the SSA1 promoter (Sup-1/Sup-4 off genomic DNA), the TFT1 ORF (Sup-3/TFT1KI-3 off genomic DNA), the Phelomycin cassette (TFT1KI-2/TFT1KI-5 off pDHT-Y-agtaΔKI), and the downstream 1kb flank of the TFT1 gene (TFT1KI-4/TFT1KI-6 off genomic DNA).

**Table 1:** 

*tft1Δ*	
Ku80-4	GTTTAGAGGTAATCCTTCTTGATCCTGAACACCATTTGTC
Ku80-5	GACAAATGGTGTTCAGGATCAAGAAGGATTACCTCTAAAC
rTFT1-1	CCACCGCGGTGGCGGCCGCTCTAGAACTAGTGGATCCCCCATGAACGGACACTATGGCCA
rTFT1-2	TGTTCCCTTCCTCACGCTGGTATAGGTCAATAGAGTATAC
rTFT1-3	GTATACTCTATTGACCTATACCAGCGTGAGGAAGGGAACA
rTFT1-4	CATTTAAGTTGAGCATAATAGATGATGTAAATGTATCGTG
rTFT1-5	CACGATACATTTACATCATCTATTATGCTCAACTTAAATG
rTFT1-6	CGACGGTATCGATAAGCTTGATATCGAATTCCTGCAGCCCTTGATGAATATGTATTCACC
**Rev*tft1*/SSA1-Rev*tft1***	
KI phleo B	AAGCTTAGCTTGCAAATTAA
KI phleo T	TCTAGACGGGTTCGCATAGG
TFT1KI-1	CCACCGCGGTGGCGGCCGCTCTAGAACTAGTGGATCCCCCACGGGGATGGTCCCTGATAA
TFT1KI-2	TGTATTCGTTCTTTTTCTGATCTAGACGGGTTCGCATAGG
TFT1KI-3	CCTATGCGAACCCGTCTAGATCAGAAAAAGAACGAATACA
TFT1KI-4	TTAATTTGCAAGCTAAGCTTGATGATGTAAATGTATCGTG
TFT1KI-5	CACGATACATTTACATCATCAAGCTTAGCTTGCAAATTAA
TFT1KI-6	CGACGGTATCGATAAGCTTGATATCGAATTCCTGCAGCCCTTGATGAATATGTATTCACC
**SSA1-Rev*tft1***	
SUP-1	TTGCCCCTCGCAAGGCCAAAGAGAGAGCCGAGGGCTACGT
SUP-2	ACGTAGCCCTCGGCTCTCTCTTTGGCCTTGCGAGGGGCAA
SUP-3	ACCCTCCTCACTTCTTCACAATGAACGGACACTATGGCCA
SUP-4	TGGCCATAGTGTCCGTTCATTGTGAAGAAGTGAGGAGGGT
**Confirmation**	
TFT1 OL-9	AAACCACACCACTTCAAAGC
TFT1 OL-10	ACCGAACTGGAGAGGTTCAT
TFT1 OL-11	GGAAGGTGTAACGTACAAGG
TFT1 OL-12	CTAAACAAGGCCGTCGAGGT
AGTAKI Left 5’	CGCTCTAACCGAAAAGGAAG
Ku80DStop	TGGCTTCACATTCTCCTTCG
Ku80USbottom	ATCTGGCATACTGTTGCTCG
SB-1	ATGAACGGACACTATGGCCA
SB-2	AGGACTTTATTCTGGAGTGC
SB-3	GACAAATGGTGTTCAGGATCAAGAAGGATTACCTCTAAAC
SB-4	CTCTGCGAGAATTTAGGCTA
SB-5	TCCTTGTCGGAGCAGCCGTT
SB-6	GTTTAGAGGTAATCCTTCTTGATCCTGAACACCATTTGTC
SB-7	AGTCGAGGAACAGACCGGCA
SB-8	TTGATGAATATGTATTCACC

**Table 2:** 

Strain Name	Description
*Af293*	*Aspergillus fumigatus* reference strain 293
*tft1Δ*	*A. fumigatus* *tft1Δ*::hph3 in Af293
*tft1Δ* (*hph3-*)	*A. fumigatus* *tft1Δ* with hph3 recycled
Rev*tft1*	*A. fumigatus* *tft1Δ* (hph3-) with *tft1* reinserted
*SSA*1-Rev*tft1*	*A. fumigatus* *tft1Δ* (hph3-) with *tft1* reinserted with SSA1 promoter
*S.c.* 6210	*Saccharomyces cervisiae* strain 6210

**Table 3:** 

Plasmid Name	Description	Reference
PSK485	β-Rec Cassette containing the upstream β-rec six recognition sequence	10
Topo-βrec/HygR	Hygromycin cassette with the downstream β-Rec six recognition sequence flanking 3' end of cassette in pCR2.1	9
pDHT-SK	*Ann tumifaciens* mediated transformation vector containing VIR ORF's for inserting tDNA	11
PDHT-Y	Modified pDHT-SK to include the Cen/Ars yeast origin of replicatio n and URA3 inserted at the Sfol restriction site	9
pDHT-Y-agtaΔKI	pDHT-Y-agtaΔKI with the agta gene and phleomycin cassette insert cloned in at the Sma1 site	Unpublished*
pDHT-Y-*tft1Δ*	pDHT-Y-*tft1Δ* with the *tft1Δ* deletion insert cloned in at the Sma1 site	This study
pDHT-Rev*tft1*	pDHT-Y-Rev*tft1* with the *tft1* gene and phleomycin cassette insert cloned in at the Sma1 site	This study
pDHT-SSA1-Rev*tft1*	pDHT-Y-Rev*tft1* with the SSA1 promoter, *tft1* gene and phleomycin cassette insert cloned in at the Sma1 site	This study
	

*Generated in our laboratory

### PCR and Southern blot analysis of tranformants.

Genomic DNA (gDNA) was prepared from fresh mycelium as previously described [9], and gDNA concentration was determined using a NanoDrop (ND100) spectrometer. For PCR analysis of the transformed strains, gDNA was amplified with primers TFT1OL-9 and 10 within the TFT1 ORF to show presence or absence of the gene. In addition, PCR was performed with primers within the resistance cassettes along with primers outside of the construct area to show expected placement of the cassette within the genome. For Southern blot, 20 μg of gDNA from each strain was digested using ScaI and SspI (NEB). Digested gDNA was separated on 0.8% agarose gel and transferred onto nitrocellulose membrane. Hybridization was achieved using a ^32^P labeled probes that spanned the entire length of the *tft1Δ* construct. Film was exposed overnight at -80°C.

### Immunofluorescence staining of cell wall glucans.

Strains were grown for 24 hours at 37°C on 8-cell chamber slides. For each strain, one slide was treated with endo β-1,3;1,4-glucanase (Megazyme) for 1 hour and washed with diluent. Primary IgG antibody against β-1,3;1,4-glucan (Biosupplies Australia) was used to detect β-1,3;1,4-glucan, with secondary antibody against mouse IgG light chain (Jackson ImmunoResearch). Images were taken with an LSM 710 microscope by Zeiss using Zen 2011 at 63x oil immersion. Z-stacks were limited to 8–10μm (~ 20 stacks). Images were processed utilizing ImageJ software [12]. All images were processed in the same manner. This experiment was repeated with antibody that specifically binds β-1,3-glucan (Biosupplies Australia) for the WT and *tft1Δ* strains, although glucanase treatment was not performed prior to β-1,3-glucan staining.

### Cell wall polysaccharide quantification.

Strains were grown for 24 hours at 37°C in Sabaroud dextrose broth with shaking at 220 RPM. Cultures were vacuum strained to collect hyphae and washed with sterile PBS. Hyphae were exhaustively disrupted using a Bead Beater (BioSpec Products) with sterile 0.5mm zirconium oxide beads. Disrupted hyphae were centrifuged at 10,000xg and the pellets were washed with NaCl and ddH_2_O 3 times each. Cell wall pellets were suspended in ddH_2_O, frozen, and lyophilized. Dry cell wall preparations were weighed. 150mg of cell walls from each strain were then incubated in 6ml 1N NaOH at 65°C for 1 hour then centrifuged as above. Soluble fractions were reserved for α-1,3-glucan quantification. Insoluble fractions were treated with 6ml 1N NaOH again. Remaining insoluble material was washed with 50mM sodium acetate, pH 5.6 to balance the pH. Samples were suspended in 6.0 ml of the sodium acetate buffer and split into 500μL aliquots. β-1,3-glucanase (Megazyme) and chitinase (Sigma) were diluted 1:10 prior to enzyme treatment. Samples were then treated exhaustively with β-1,3;1,4-glucanase (Megazyme), β-1,3-glucanase, or chitinase for 1 week at 37°C. For each digestion, three replicates were performed. Reducing sugar assay (4-hydroxybenzhydrazide method) was performed as previously described on each sample to evaluate the amount of reducing sugar released by the enzyme, thus estimating the β-1,3;1,4-glucan, β-1,3-glucan, and chitin content in each cell wall preparation [13]. For α-1,3 glucan quantification, 1 ml of soluble fractions reserved from above were analyzed. 200uL of 5N HCL was added to each tube to precipitate the soluble fraction. Pellets were washed with sodium acetate buffer. Samples were treated with α-1,3-glucanase (Megazyme) and incubated at 37°C overnight. Reducing sugar assay was performed as above on each sample to estimate the relative α-1,3-glucan content in each cell wall preparation [13]. Sigma-Plot using One Way ANOVA analysis was used to calculate significance between strains.

### Assessment of in vitro growth.

Serial dilutions of strains were created at 10^6^, 10^5^, and 10^4^ spores per ml. 5 μl of each dilution (i.e. 5000, 500, 50 spores) from each strain was plated onto various media, including *Aspergillus* minimal media (AMM), and AMM containing Farnesol (25μg/mL), Calcoflour White (100μg/mL) or Congo Red (55μg/mL) [14]. Plates were grown for 2 days at 37°C and then evaluated for any growth phenotypes. In addition, spores and hyphae from each strain were subjected to cold conditions (4°C) prior to either inoculation onto AMM and incubation at 37°C (for spores) or moving from the cold conditions to 37°C (for hyphae) to assess the effects of cold incubation on subsequent growth.

### Antifungal susceptibility.

Antifungal susceptibility was tested for each strain using standard methods [15]. RPMI 1640 with HEPES (Invitrogen) was used as the medium for the assay. Caspofungin and Nikkomycin (Sigma) were used in concentrations from 64μg/mL to 0.0625μg/mL via serial dilution. 100μL of 1.0x10^4^ spores/ml were used to inoculate each well. Growth was assessed visually. Concurrently, MM agar plates with either 1.0 μg/mL of Caspofungin or Nikkomycin were inoculated for observation of radial growth at 37°C for 3–5 days.

### Galleria Mellonella challenge.

The Galleria worm model of *A. fumigatus* virulence was performed as previously described [16]. Briefly, spores suspensions of each strain were made at 1.0x10^8^ and 2.0x10^8^ spores/mL in 0.01M PBS. *G. mellonella* (Vanderhorst Wholesale) worms were chosen by weight at 0.2–0.4g and placed in a petri dish with a small amount of wood chips at 15 worms per dish. Hamilton 10μL syringes were used to inject 5.0μL of desired spore suspension into the left proleg of each worm [16]. Worms were incubated at 37°C and observed daily for mortality over a 5 day period. Dead worms were determined by the lack of response to tactile stimulation and removed from the plate. The *acumΔ* control strain was a kind gift from Scott Filler [17]. SigmaPlot was utilized to analyze data via Kaplan-Meier survival-log rank analysis.

## Results

### Homology searches and topology.

*CSLF* and *CSLH* are plant enzymes that have been shown to have β-1,3;1,4 glycosyltransferase activity in rice and barley, respectively. As such, *A. fumigatus* orthologs, if present, would potentially have similar activity and likely involved in cell wall synthesis. The two plant protein sequences were blasted against the *A. fumigatus* database in NCBI. Only one protein in the *A. fumigatus* database appeared to have any homology to these two enzymes. This protein (XP_748682/Afu3g03620), which was renamed *Tft1*, was annotated in NCBI as a putative glycosyltransferase. There was only about 30% homology between either plant sequence and *Tft1*, but the homology present was confined entirely within the “CesL” (Cellulose Synthase-like) domains of both plant sequences. CesL is the putative active site of the CSL glycosyltransferase enzymes [7,8]. In addition, to determine if there were orthologs to *Tft1* in other organisms, the *Tft1* protein sequence was blasted back into the general NCBI database. Interestingly, there were close orthologs (70–95% identity) in other fungal organisms. All the organisms with orthologs were not only fungi, but all were ascomycetes. These included Neosartorya (the sexual anamorph of *A. fumigatus*), other species of *Aspergillus* including *A. flavus* and *A. clavatus*, *Fusarium oxysporum*, and numerous species of *Trichoderma* and *Metarhizium*. Finally, the protein sequence of *Tft1* was submitted to transmembrane prediction software (TMPRED: http://www.ch.embnet.org/software/TMPRED_form.html). It is predicted to be a 7 pass transmembrane protein, a common feature of many membrane bound glycosyltransferases in fungi [18].

### Strain generation and confirmation.

Three strains were developed using *Agrobacterium tumefaciens* Mediated Transformation (ATMT). One strain with the *Tft1* gene deleted (*tft1Δ*) and two additional revertant strains where the native *Tft1* gene was added back to the *tft1Δ* tranformant under control of either the native promoter (Rev*tft1*) or the strongly constitutive SSA1 promoter (SSA1-Rev*tft1*) [19]. Strain confirmations were achieved by PCR and Southern Blot. PCR was used to amplify the *Tft1* gene in each strain. Af293, Rev*tft1* and SSA1-Rev*tft1* were all positive for the presence of the *Tft1* gene, while the *tft1Δ* strain was negative for the *Tft1* gene (S1 Fig.). To confirm proper insertion of each construct, PCR was performed with a primer outside the construct integration site, coupled with a primer within the construct. All strains had the correct amplification patterns and were determined to have the proper orientation at the site of integration (S1 Fig.). Southern blot was used to determine that only a single, correct integration event was present throughout the genome. Genomic preparations from each strain were digested with restriction enzymes SspI and ScaI, which allowed for the differentiation between the strains with probes aimed at the *tft1Δ* construct. As shown in S2 Fig., all strains presented the correct banding patterns without any additional, unexpected bands.

### Immunofluorescence staining of β-1,3;1,4-glucan.

**Fig 1:**
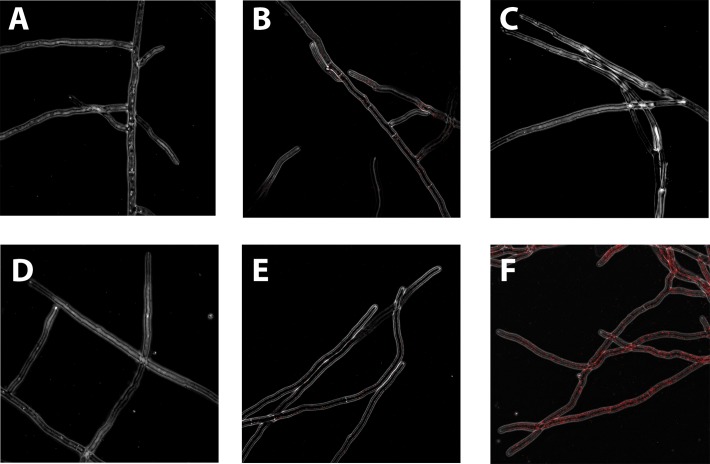
Immunostaining of hyphae with a specific β-1,3;1,4 glucan antibody. A. No primary antibody Af293(WT) control. β-1,3;1,4-glucan staining of B. WT.; C. β-1,3;1,4-glucanase digested WT(only WT shown; other digested strains were also negative for staining); D. *tft1Δ*; E. Rev*tft1*; and F. SSA1Rev*tft1*.

Immunofluorescence staining of β-1,3;1,4-glucan on the surface of each strain was performed using an antibody. This antibody has been shown in numerous studies in plants to be specific for this β-1,3;1,4 glucan, with no binding to β-1,3 glucan or other plant cell wall polysaccharides [6,20]. As shown in Fig. 1, in WT (Af293), β-1,3;1,4-glucan staining was present throughout the hyphae in a speckled pattern. On rare occasion, fruiting structures were observed (expected to be rare in the growth conditions used). The fruiting structures stained more brightly than hyphae with this antibody. Pre-treatment of WT hyphae with β-1,3;1,4-glucanase prior to immunostaining abrogated the WT staining. Pretreatment of the other strains yielded the same negative result. Consistent with the proposed role of *Tft1*, the hyphae from the *tft1Δ* strain had no staining, appearing identical to results from the no primary antibody control and the β-1,3;1,4-glucanase digestion samples. Reintroduction of the WT gene under the control of the native promoter (Rev*tft1*) yielded staining very similar to the WT strain. When TFT1 was replaced under the control of the strong, constitutive SSA1 promoter, the immunostaining was considerably increased across the entire surface of the hyphae (Fig. 1). Staining was also performed on the WT and *tft1Δ* strains with an antibody that specifically binds β-1,3 glucan. Very strong staining was observed for both strains, preventing observation of subtle differences between the strains for this polysaccharide (S3 Fig.).

### Cell wall polysaccharide quantification.

**Fig 2:**
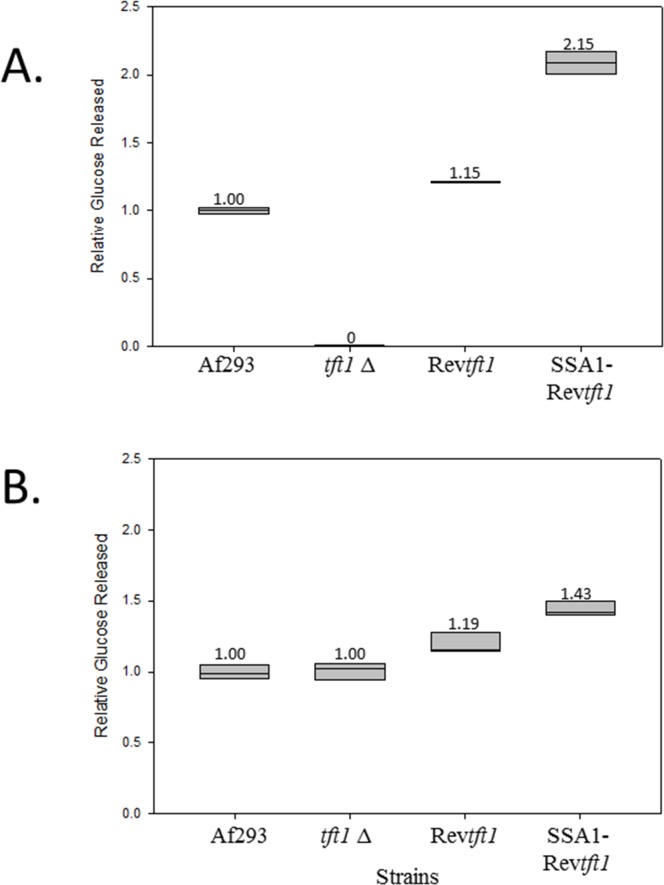
The relative glucose released when insoluble cell wall fractions were digested with A. endo β-1,3;1,4-glucanase or B. β-1,3 glucanase. *tft1Δ* shown to release zero glucose when exposed to endo β-1,3;1,4-glucan hydrolase. The box represents calculated error based on replicates of 3 samples for each digestion. WT vs *tft1Δ*, p<0.001; WT vs Rev*tft1*, p<0.001; WT vs SSA1-Rev*tft1*, p<0.001.

In order to biochemically quantify the relative amount of a cell wall polysaccharide, a common strategy is to specifically enzymatically digest the polysaccharide in cell wall preparations and measure the amount of new reducing ends formed by the specific enzymatic digestion [13]. This was done for all four strains on the alkali insoluble fraction of the cell wall using a specific endo β-1,3;1,4 glucanase [21], β-1,3 glucanase, and chitinase. Equal masses of the cell wall preparations from all four strains were extracted exhaustively with sodium hydroxide. The alkali soluble portion was reserved and later used subsequently to quantify α-1,3-glucan as a loading control. The alkali insoluble portion derived from each strain was then split and either left untreated or exhaustively digested with the β-1,3;1,4-glucanase, β-1,3 glucanase, or chitinase. As shown in Fig. 2A, the pattern of relative β-1,3;1,4 glucan observed in the cell wall of each strain correlates well with the immunostaining results. Cell walls from *tft1Δ* had no observable new reducing ends formed after digestion. The revertant Rev*tft1* strain with replacement of TFT1 with the native promoter had a relative glucose release of 115% that of the parent Af293 strain; and SSA1-rev*tft1* with expression driven by the strong SSA1 promoter had a relative glucose release of 215% that of Af293. As shown in Fig. 2B, β-1,3 glucanase digestion demonstrated an increase in glucose release in rev*tft1* and SSA1 -rev*tft1*, while *tft*1∆ demonstrated no change when compared to Af293. No significant difference in glucose release was observed between any of the four strains after treatment with chitinase (S3 Fig.). To ensure equal amounts of cell wall preparations were used for each strain in the β-1,3;1,4-glucan quantification, the alkali soluble fraction from above was treated with α-1,3-glucanase and evaluated in the same manner. After overnight incubation, all strains were shown to be roughly equal in reducing ends formed by this enzyme (S4 Fig.). Considering that this cell wall component is in a different cell wall compartment and is most likely quantitatively unrelated to the insoluble fraction, this suggests that the cell wall input was similar for all four strains.

### Assessment of *in vitro* growth.

**Fig 3:**
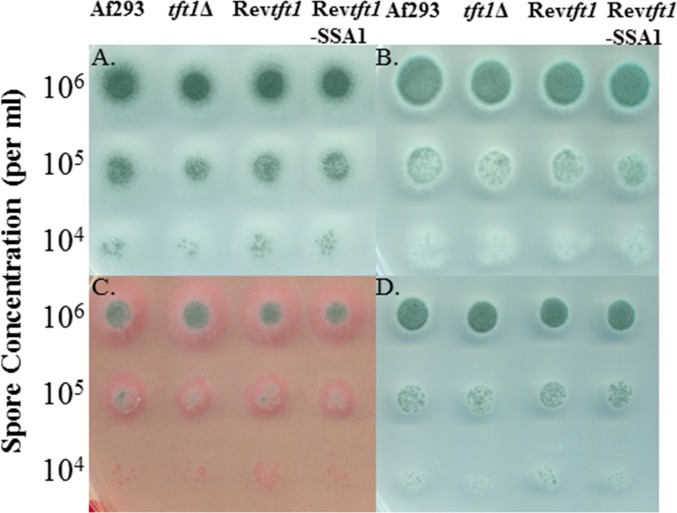
In vitro growth of the *Tft1* transformant strains. Strains grown on, A. *Aspergillus* Minimal Media (AMM); B. AMM+Farnesol; C. AMM+Congo Red; or D AMM-Calcofluor white. No apparent differences in phenotype were observed between the strains and WT.

As shown in Fig. 3, both sporulation and radial growth were identical for all four strains on *Aspergillus* Minimal Media (AMM). Addition of farnesol, an inhibitor of the cell wall integrity pathway [22], into the AMM plates resulted in a slight decrease in sporulation and radial growth, but this effect was observed equally across all strains. Addition of the cell wall stress agents Congo red or calcofluor white into the AMM plates resulted in significant stress on all colonies, but no phenotypes were observed for any of the *tft1* strains. In addition, both spores and hyphae from the strains were subjected to cold conditions prior to incubation at 37°C. Regardless of the cold incubation time or if hyphae or spores were subjected to cold, there were no differences between the strains in tolerating cold incubation (not shown).

### Antifungal Susceptibility.

In order to determine the effect of cell wall changes on antifungal susceptibility, minimum effective concentration (MEC) was determined for two cell wall active antifungal agents. Nikkomycin, an inhibitor of chitin synthesis, was shown to have no effect on any strain at any concentration. Caspofungin was shown to have a minimum effective concentration of .0625μg/mL for all strains tested. Wells containing concentrations above .0625μg/mL of caspofungin demonstrated increased growth inhibition. However, no visible differences were observed between strains in susceptibility to this β-1,3 glucan synthase inhibitor.

### *Galleria Mellonella* challenge.

**Fig 4:**
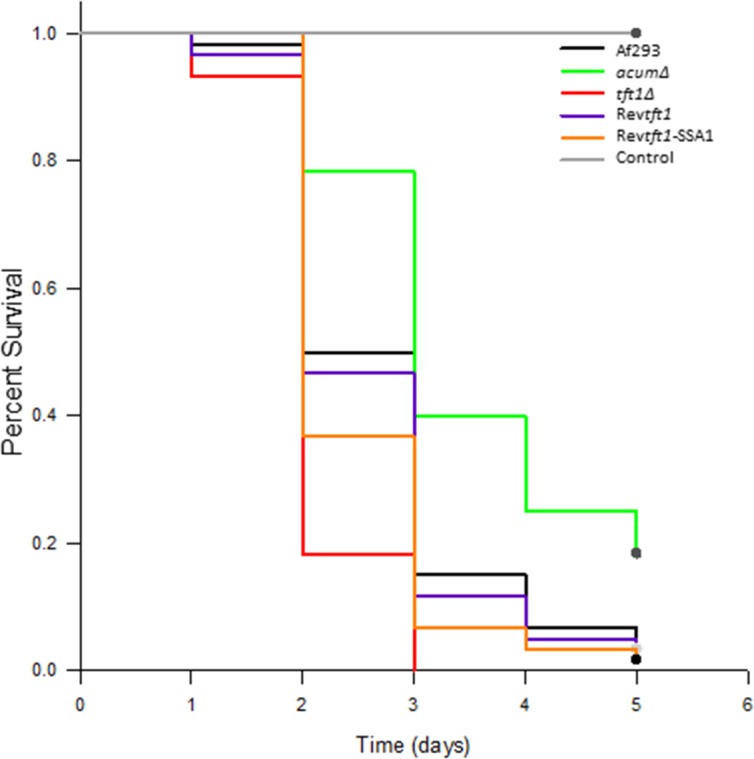
Log rank survival analysis of each strain after 1 x 10^6^ spore injection into *Galleria mellonella*. *tft1Δ* was shown to have a slight, yet significant (p value: .000187), increase in virulence when compared to its parent Af293. This hyper-virulence was lost upon re-introduction of the gene, either under native or strong promoter (Rev*tft1* and SSA1Rev*tft1*).

To gain a general understanding of the effect of loss of mixed linkage glucan on virulence, the increasingly popular Galleria model was utilized [17,23]. For each strain tested, 60 worms were injected with 1 x 10^6^ spores, and the worms were observed over 5 days for mortality. As shown in Fig. 4, the placebo group that was injected with diluent survived all 5 days of the experiment with no signs of morbidity. The *acumΔ* strain is a known hypovirulent strain in this model [17] and was used as a hypovirulent control. Morbidity for this control strain was observed on days 3–5, with some worms surviving, as compared to WT (Af293) for which most worms were dead by day 4 and nearly all dead by day 5. Worms injected with *tft1Δ* were visibly sick by day 1, and the group was completely dead by day 2/3. This hypervirulent phenotype was significant compared to WT (p-value: .00019). Rev*tft1* and SSA1-rev*tft1* were shown to have no significant virulence difference as compared to WT.

## Discussion

*Aspergillus fumigatus* is an important filamentous fungal pathogen that can cause life threatening infections in immunocompromised patients. New treatment options for invasive fungal infections, including aspergillosis, are a key need. The cell wall is a structure that is essential for the survival of fungi, and humans do not make an analogous structure. However, while cell wall synthesis represents a promising area to find new anti-fungal drug targets, our understanding of how this structure is built, especially in molds, is crude. The cell wall of *A. fumigatus* is primarily composed of various cross-linked polysaccharides, including a novel mixed linkage glucan [4]. This β-1,3;1,4 glucan represents approximately 10% of the *A. fumigatus* cell wall glucan content, but the roles that this molecule plays for this mold are unknown [5]. Further, the enzyme(s) that synthesize this polysaccharide in *A. fumigatus* are also not known. Mixed linkage glucan is also found in various plants, and it is an important molecule in the agricultural industry, as it is undesirable for various downstream food processing applications [6]. As such, there has been great interest in finding the enzyme that makes this molecule in plants, and there are specific commercially available reagents to detect the presence of mixed linkage glucan. The identity of two such mixed linkage glucan synthases in rice and barley were discovered in 2006 and 2009, respectively [7,8]. Using the sequence of these two enzymes, we found one ortholog in the *A. fumigatus* genomic database. While this ortholog had only minimal homology with the two plant sequences, the relatively short stretch of homology was completely within the putative active site of the plant enzymes [7,8]. We named this putative synthase *Tft1* for Three-four transferase 1. This was annotated as a predicted glycosyltransferase and the TMPRED transmembrane prediction software predicted the sequence to have seven transmembrane passes. The predicted transmembrane localization is consistent with a potential role in cell wall synthesis, as enzymes shown to synthesize cell wall polysaccharides are commonly localized to membranes [18]. Using our recently published gene deletion system for *A. fumigatus* [9], we deleted the *Tft1* gene. With the same system, we also replaced the WT *Tft1* gene in the deletion strain under the control of either the WT or a strongly expressing promoter. Loss of the gene led to complete loss of β-1,3;1,4 glucan in the cell wall as detected by both immunofluorescence and biochemical quantitation.

Staining of WT hyphae with an antibody specific to β-1,3 glucan yields smooth, linear staining of the entire hyphal surface. In contrast, the pattern of immunofluorescence staining in the WT strain using the antibody specific to β-1,3;1,4 glucan was consistently punctate and relatively weak. This could be due to the comparatively lower prevalence of the molecule in the cell wall and/or the length of β-1,3;1,4 glucan needed for antibody binding. In addition, we also observed much brighter staining on the rarely encountered conidiophores, suggesting that this molecule might play a role in spore formation. While staining of spore preparations was negative, this is not unexpected due to the outer rodlet protein layer on spores that prevents antibody binding to the polysaccharides in the wall beneath [24]. Finally, significantly increased staining of hyphae with the β-1,3;1,4 glucan antibody was observed in the strain overexpressing TftT1. This was reinforced by a 2-fold increase of this molecule when biochemically quantified within the cell wall. As above, this further supports the direct role of this enzyme in the synthesis of the cell wall mixed linkage glucan.

Another interesting observation was that the β-1,3 glucan content in the cell wall appeared to increase in the strain overexpressing β-1,3;1,4 glucan. This could merely represent some digesting of the increased mixed linkage glucan with the β-1,3 glucanase. However, if there is a role for mixed linkage glucan in triggering or anchoring for β-1,3 glucan synthesis, this would also potentially be observed. Unfortunately, there are not good experimental tools to currently test this possibility.

In vitro growth of the *tft1Δ* strain was essentially the same as both WT and the strain overexpressing the gene on multiple media types and under a number of various conditions, including in the presence of cell wall stress agents or cell wall active antifungals. This would strongly suggest that cell wall β-1,3;1,4 glucan is dispensable for the growth and fitness of *A. fumigatus* and that a 2-fold excess of the molecule is not deleterious, either. Even though this enzyme does not appear to be a suitable antifungal drug target, we did assess virulence in the strains. Virulence was assessed with the *Galleria mellonella* wax worm model [16]. Interestingly, the loss of *Tft1*, and thus mixed linkage glucan, resulted in a slight, albeit significant, hyper-virulent phenotype. Increase of the molecule in the overexpressing strain had the same virulence pattern as WT, suggesting the specific glucanases that degrade this molecule would also not likely be suitable therapeutic targets. While the synthesis or degradation of mixed linkage glucan does not appear to harbor a promising drug target, this does represent the first report of a β-1,3;1,4 glucan synthase in fungi.

## Supporting Information

S1 Fig PCR confirmation of each TFT1 transformant. A. Af293 *tft1* gene amplification positive with expected band size appearing at 500bp. B. *tft1Δ* gene amplification negative with no band at expected 500bp. C. *tft1Δ* upstream flank amplification positive with expected band size appearing at 1800bp. D. *tft1Δ* downstream flank amplification positive with expected band size appearing at 1300bp. E. Rev*tft1* gene amplification positive with expected band size appearing at 500bp. F. Rev*tft1* upstream flank amplification positive with expected band size appearing at 2500bp. G. Rev*tft1* downstream flank amplification positive with expected band size appearing at 1300bp. H. SSA1-Rev*tft1* gene amplification positive with expected band size appearing at 500bp. I. SSA1-Rev*tft1* upstream flank amplification positive with expected band size appearing at 3400bp. J. SSA1-Rev*tft1* downstream flank amplification positive with expected band size appearing at 1300bp.
(TIF)

S2 Fig Southern blot confirmation of each *Tft1* transformant. A. Expected band size of 6750bp appeared for Af293. B. Expected band size pattern of 4990bp, 2240bp, 1920bp, and 1083bp appeared for *tft1Δ*. C. Expected band size pattern of 4900bp and 3490bp appeared for Rev*tft1*. Expected band size pattern of 4899bp and 4940bp (seen as a doublet) appeared for SSA1-Rev*tft1*.
(TIF)

S3 Fig Immunostaining of hyphae with a specific β-1,3-glucan antibody. (TIF)

S4 Fig Relative reducing glucose released with chitinase. Cell wall preparations from each strain were exhaustively digested with chitinase and then quantitatively assayed for reducing sugar released. There was no difference among the four strains. The box represents the range of values observed based on replicates of 3 samples for each digestion, with the line being the average. There was no signficant difference between WT and *tft1Δ*.
(TIF)

S5 Fig Relative reducing glucose released with α-1,3 glucanase. Cell wall preparations from each strain were exhaustively digested with α-1,3 glucanase and then quantitatively assayed for reducing sugar released. There was no statistical difference among the four strains. The box represents the range of values observed based on replicates of 3 samples for each digestion, with the line being the average.
(TIF)

## References

[1] Kontoyiannis DP, Marr KA, Park BJ, Alexander BD, Anaissie EJ, et al (2010) Prospective surveillance for invasive fungal infections in hematopoietic stem cell transplant recipients, 2001–2006: overview of the Transplant-Associated Infection Surveillance Network (TRANSNET) Database. Clin Infect Dis 50: 1091–1100. 10.1086/651263 20218877

[2] Baddley JW (2011) Clinical risk factors for invasive aspergillosis. Med Mycol 49 Suppl 1: S7–S12. 10.3109/13693786.2010.505204 20718606

[3] Bernard M, Latge JP (2001) *Aspergillus fumigatus* cell wall: composition and biosynthesis. Med Mycol 39 Suppl 1: 9–17. 11800273

[4] Fontaine T, Simenel C, Dubreucq G, Adam O, Delepierre M, et al (2000) Molecular organization of the alkali-insoluble fraction of *Aspergillus fumigatus* cell wall. J Biol Chem 275: 27594–27607. 10869365 10.1074/jbc.M909975199

[5] Latge JP, Mouyna I, Tekaia F, Beauvais A, Debeaupuis JP, et al (2005) Specific molecular features in the organization and biosynthesis of the cell wall of *Aspergillus fumigatus* . Med Mycol 43 Suppl 1: S15–22. 16110787 10.1080/13693780400029155

[6] Meikle PJ, Hoogenraad NJ, Bonig I, Clarke AE, Stone BA (1994) A (1—>3,1—>4)-beta-glucan-specific monoclonal antibody and its use in the quantitation and immunocytochemical location of (1—>3,1—>4)-beta-glucans. Plant J 5: 1–9. 8130794 10.1046/j.1365-313x.1994.5010001.x

[7] Burton RA, Wilson SM, Hrmova M, Harvey AJ, Shirley NJ, et al (2006) Cellulose synthase-like *CslF* genes mediate the synthesis of cell wall (1,3;1,4)-beta-D-glucans. Science 311: 1940–1942. 16574868 10.1126/science.1122975

[8] Doblin MS, Pettolino FA, Wilson SM, Campbell R, Burton RA, et al (2009) A barley cellulose synthase-like *CSLH* gene mediates (1,3;1,4)-beta-D-glucan synthesis in transgenic Arabidopsis. Proc Natl Acad Sci U S A 106: 5996–6001. 10.1073/pnas.0902019106 19321749 PMC2667043

[9] Kieler JB, Duong KL, Moye-Rowley WS, Klutts JS (2013) Targeted gene deletion in *Aspergillus fumigatus* using microbial machinery and a recyclable marker. J Microbiol Methods 95: 373–378. 10.1016/j.mimet.2013.09.021 24161898

[10] Hartmann T, Dumig M, Jaber BM, Szewczyk E, Olbermann P, et al (2010) Validation of a self-excising marker in the human pathogen *Aspergillus fumigatus* by employing the beta-rec/six site-specific recombination system. Appl Environ Microbiol 76: 6313–6317. 10.1128/AEM.00882-10 20656854 PMC2937505

[11] Sugui JA, Chang YC, Kwon-Chung KJ (2005) *Agrobacterium tumefaciens*-mediated transformation of *Aspergillus fumigatus*: an efficient tool for insertional mutagenesis and targeted gene disruption. Appl Environ Microbiol 71: 1798–1802. 15812003 10.1128/AEM.71.4.1798-1802.2005 PMC1082565

[12] Schneider CA, Rasband WS, Eliceiri KW (2012) NIH Image to ImageJ: 25 years of image analysis. Nat Methods 9: 671–675. 22930834 10.1038/nmeth.2089 PMC5554542

[13] Mouyna I, Morelle W, Vai M, Monod M, Lechenne B, et al (2005) Deletion of GEL2 encoding for a beta(1–3)glucanosyltransferase affects morphogenesis and virulence in *Aspergillus fumigatus* . Mol Microbiol 56: 1675–1688. 15916615 10.1111/j.1365-2958.2005.04654.x

[14] Ram AF, Klis FM (2006) Identification of fungal cell wall mutants using susceptibility assays based on Calcofluor white and Congo red. Nat Protoc 1: 2253–2256. 17406464 10.1038/nprot.2006.397

[15] Clinical and Laboratory Standards Institute Antifungal Testing S (2008) Reference Method for Broth Dilution Antifungal Susceptibility Testing of Filamentous Fungi; Approved Standard-Second Edition. M38-A2.

[16] Fuchs BB, O'Brien E, Khoury JB, Mylonakis E (2010) Methods for using Galleria mellonella as a model host to study fungal pathogenesis. Virulence 1: 475–482. 21178491 10.4161/viru.1.6.12985

[17] Liu H, Gravelat FN, Chiang LY, Chen D, Vanier G, et al (2010) *Aspergillus fumigatus* AcuM regulates both iron acquisition and gluconeogenesis. Mol Microbiol 78: 1038–1054. 10.1111/j.1365-2958.2010.07389.x 21062375 PMC3051834

[18] Klutts JS, Yoneda A, Reilly MC, Bose I, Doering TL (2006) Glycosyltransferases and their products: cryptococcal variations on fungal themes. FEMS Yeast Res 6: 499–512. 16696646 10.1111/j.1567-1364.2006.00054.x

[19] Paul S, Klutts JS, Moye-Rowley WS (2012) Analysis of promoter function in *Aspergillus fumigatus* . Eukaryot Cell 11: 1167–1177. 10.1128/EC.00174-12 22843562 PMC3445982

[20] Brown RC, Lemmon BE, Stone BA, Olsen OA (1997) Cell wall (1—>3)- and (1—>3, 1—>4)-beta-glucans during early grain development in rice (Oryza sativa L.). Planta 202: 414–426. 9265785 10.1007/s004250050145

[21] McCleary BV, Shameer I, Glennie-Holmes M (160) Measurement of (1–3), (1–4)-Beta-D-Glucan. Methods Enzymol 160: 511–514.

[22] Dichtl K, Ebel F, Dirr F, Routier FH, Heesemann J, et al (2010) Farnesol misplaces tip-localized Rho proteins and inhibits cell wall integrity signalling in *Aspergillus fumigatus* . Mol Microbiol 76: 1191–1204. 10.1111/j.1365-2958.2010.07170.x 20398212

[23] Gravelat FN, Ejzykowicz DE, Chiang LY, Chabot JC, Urb M, et al (2010) *Aspergillus fumigatus* MedA governs adherence, host cell interactions and virulence. Cell Microbiol 12: 473–488. 10.1111/j.1462-5822.2009.01408.x 19889083 PMC3370655

[24] Aimanianda V, Bayry J, Bozza S, Kniemeyer O, Perruccio K, et al (2009) Surface hydrophobin prevents immune recognition of airborne fungal spores. Nature 460: 1117–1121. 10.1038/nature08264 19713928

