# Supplementary figures and images for: Identification and Deletion of Tft1, a Predicted Glycosyltransferase Necessary for Cell Wall β-1,3;1,4-Glucan Synthesis in Aspergillus fumigatus
Source: PLoS One. 2015 Feb 27;10(2):e0117336. doi: 10.1371/journal.pone.0117336 (PMC4344333; doi:10.1371/journal.pone.0117336)

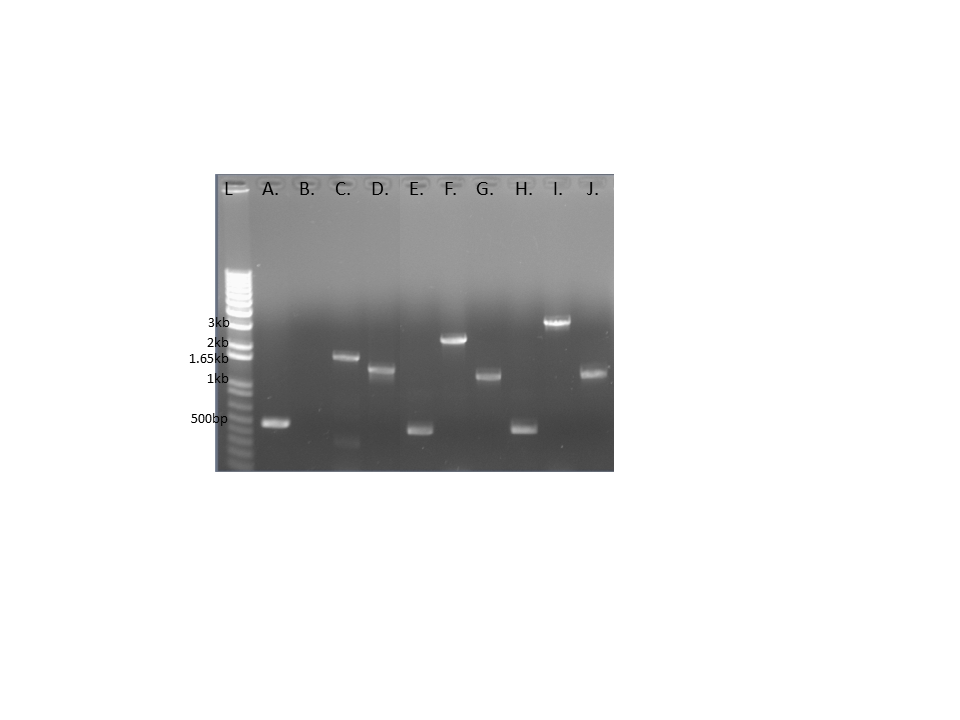

Supplement: S1 Fig — A. Af293 tft1 gene amplification positive with expected band size appearing at 500bp. B. tft1Δ gene amplification negative with no band at expected 500bp. C. tft1Δ upstream flank amplification positive with expected band size appearing at 1800bp. D. tft1Δ downstream flank amplification positive with expected band size appearing at 1300bp. E. Revtft1 gene amplification positive with expected band size appearing at 500bp. F. Revtft1 upstream flank amplification positive with expected band size appearing at 2500bp. G. Revtft1 downstream flank amplification positive with expected band size appearing at 1300bp. H. SSA1-Revtft1 gene amplification positive with expected band size appearing at 500bp. I. SSA1-Revtft1 upstream flank amplification positive with expected band size appearing at 3400bp. J. SSA1-Revtft1 downstream flank amplification positive with expected band size appearing at 1300bp. (TIF) [file pone.0117336.s001.tif]

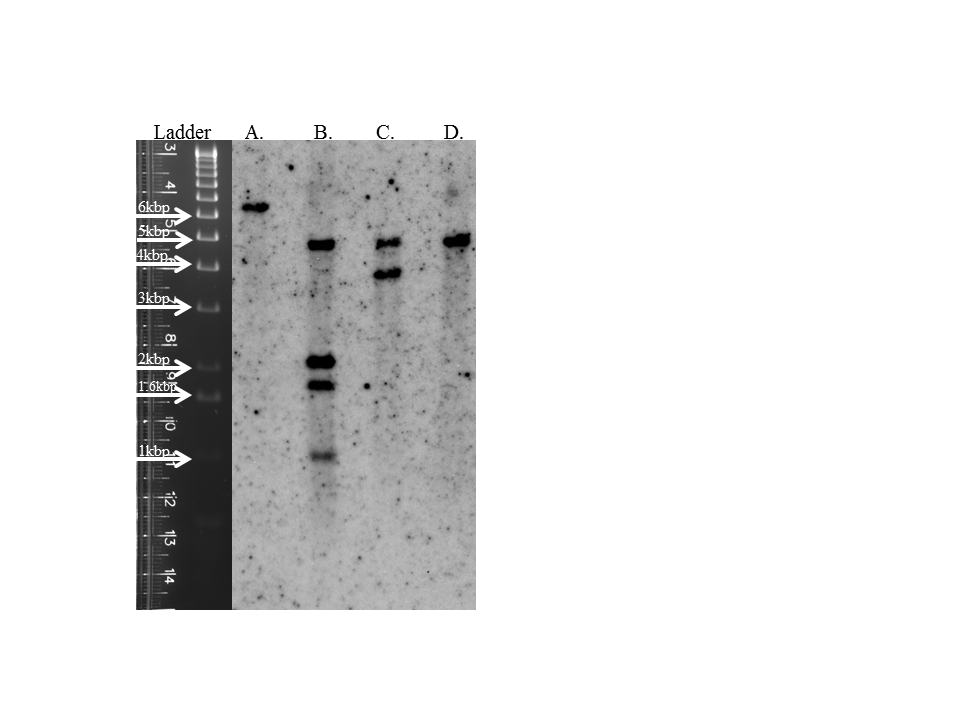

Supplement: S2 Fig — A. Expected band size of 6750bp appeared for Af293. B. Expected band size pattern of 4990bp, 2240bp, 1920bp, and 1083bp appeared for tft1Δ. C. Expected band size pattern of 4900bp and 3490bp appeared for Revtft1. Expected band size pattern of 4899bp and 4940bp (seen as a doublet) appeared for SSA1-Revtft1. (TIF) [file pone.0117336.s002.tif]

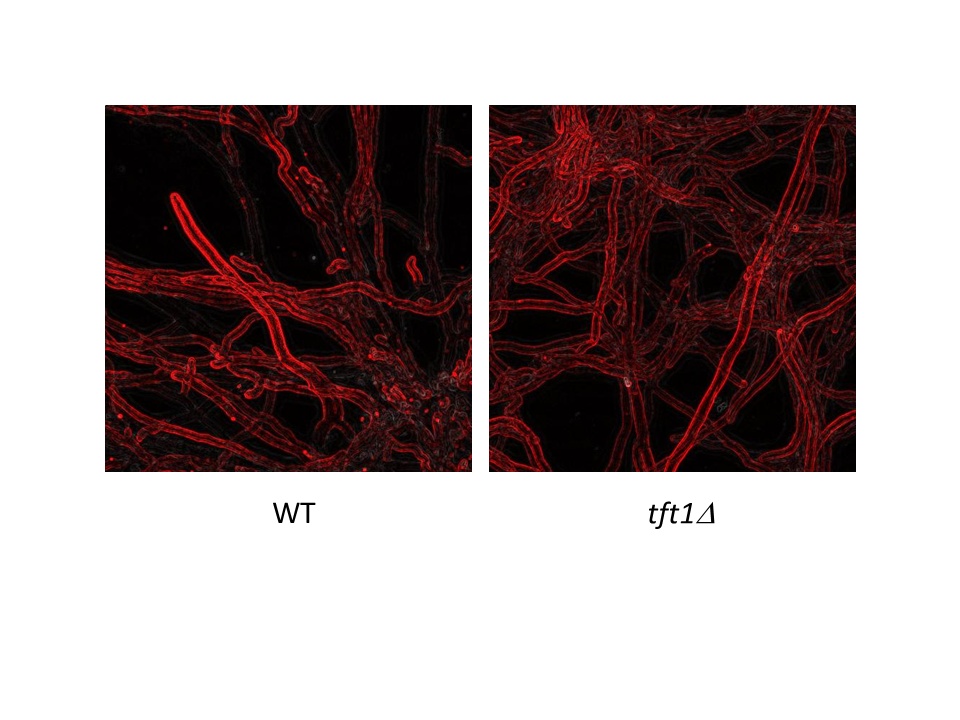

Supplement: S3 Fig — (TIF) [file pone.0117336.s003.tif]

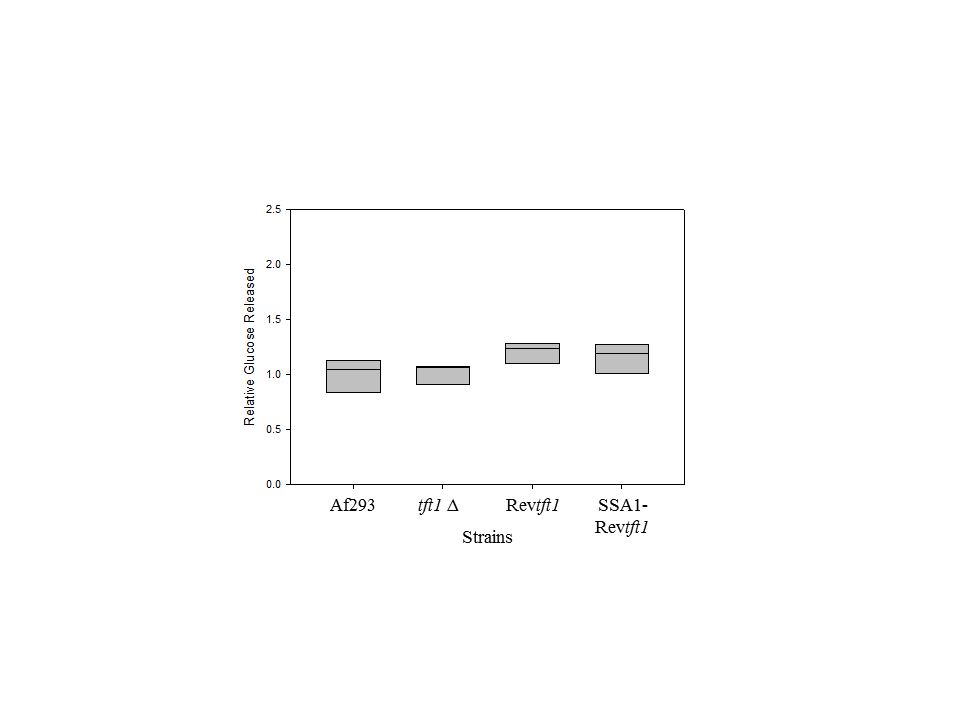

Supplement: S4 Fig — Cell wall preparations from each strain were exhaustively digested with chitinase and then quantitatively assayed for reducing sugar released. There was no difference among the four strains. The box represents the range of values observed based on replicates of 3 samples for each digestion, with the line being the average. There was no signficant difference between WT and tft1Δ. (TIF) [file pone.0117336.s004.tif]

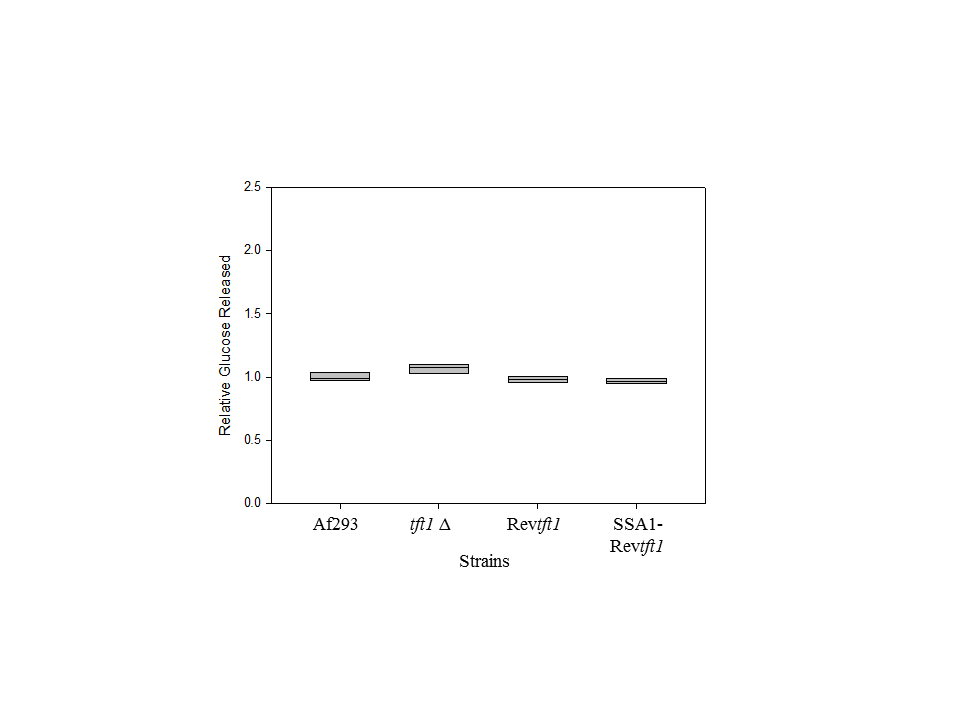

Supplement: S5 Fig — Cell wall preparations from each strain were exhaustively digested with α-1,3 glucanase and then quantitatively assayed for reducing sugar released. There was no statistical difference among the four strains. The box represents the range of values observed based on replicates of 3 samples for each digestion, with the line being the average. (TIF) [file pone.0117336.s005.tif]
